# Mitigation of letrozole induced polycystic ovarian syndrome associated inflammatory response and endocrinal dysfunction by *Vitex negundo* seeds

**DOI:** 10.1186/s13048-024-01378-4

**Published:** 2024-04-08

**Authors:** Tarun Kumar Kar, Sananda Sil, Angshita Ghosh, Ananya Barman, Sandip Chattopadhyay

**Affiliations:** Department of Biomedical Laboratory Science & Management, Vidyasagar University, Midnapore, West Bengal India

**Keywords:** Polycystic ovarian syndrome, *Vitex Nigundo* ethanolic extract, Letrozole, NR3C4, ERα, Androgen, Inflammation

## Abstract

**Background:**

Polycystic ovary syndrome (PCOS) is a complex endocrine disorder in women that necessitates effective and safe treatment alternatives. This study aimed to evaluate the therapeutic efficacy of *Vitex negundo* seed in a letrozole-induced PCOS rat model.

**Results:**

Findings of the present study demonstrated that administration of hydro-ethanolic extract of *Vitex negundo* (VNE) effectively restored endocrino-metabolic imbalances associated with PCOS, along with correction of antioxidant enzymes level, proinflammatory cytokines, and apoptotic bio-markers. LC-MS analysis confirmed the presence of cinnamic acid, plumbagin and nigundin B as the prominent phytochemicals in VNE. The observed beneficial effects could be attributed to the active compounds in *Vitex negundo* extract, which exhibited hypoglycemic, hypolipidemic, and catabolic effects on body weight. Additionally, the extract contributed to hormonal balance regulation by modulating the steroidogenic enzymes, specifically by tuning gonadotropins level and correcting the LH:FSH ratio, through the modulation of ERα signalling and downregulation of NR3C4 expression. The antioxidant properties of phytochemicals in *Vitex negundo* seed were apparent through the correction of SOD and catalase activity. While it’s anti-inflammatory and antiapoptotic action were associated with the regulation of mRNA expression of TNF-α, IL-6, BAX, Bcl2. Molecular docking study further indicated the molecular interaction of above mentioned active phytocompounds of VNE with ERα, NR3C4 and with TNFα that plays a critical mechanistic gateway to the regulation of hormone signalling as well as synchronizing the inflammation cascade. Furthermore, the histomorphological improvement of the ovaries supported the ameliorative action of *Vitex negundo* extract in the letrozole-induced PCOS model.

**Conclusions:**

This study indicates the potential of *Vitex negundo* seed as a multifaceted therapeutic option for PCOS. VNE offers a holistic strategy for PCOS with antiandrogenic, anti-inflammatory, and antioxidant properties, driven by its major compounds like cinnamic acid, plumbagine, and nigundin B.

## Background

Polycystic ovary syndrome (PCOS) is a heterogeneous endocrine disorder, affecting approximately 5–18% of reproductive-aged women worldwide [1]. The etiology of PCOS is not well understood but is thought to involve genetic, environmental, and lifestyle factors [2]. PCOS is characterized by a combination of symptoms, including menstrual dysfunction, hyperandrogenism, and polycystic ovaries. Numerous studies showed an elevated inflammation in both the reproductive organs and systemic levels in individuals with PCOS [3]. Ovarian inflammation results in poor oocyte quality, oligo-anovulation and ultimately infertility [3]. Concurrently, chronic low-grade systemic inflammation in PCOS interacts with hyperandrogenism, obesity, and insulin resistance, precipitating metabolic disturbances and increasing the risk of Type 2 diabetes and cardiovascular disease [4]. Therefore, the goals of PCOS treatment are to rectify inflammation, ovulatory dysfunction, reduce androgen level, manage body weight, and mitigate the long-term risks of metabolic non-communicable disease [5].

Current medical interventions for PCOS management mainly focuses on symptomatic temporary relieves and often causing undesirable side effects. The commonly used medical strategy for PCOS encourages the use of oral contraceptive pills, anti-androgens spironolactone, insulin-sensitizing drug metformin, fertility medications like clomiphene citrate, and laparoscopic ovarian drilling. However, these therapies are associated with limited efficacy and may not provide satisfactory outcomes [6].

The limitations and adverse effects of conventional therapies for PCOS lead to a growing interest in exploring safe alternative treatment options. Medicinal plants and their phytoconstituents have gained attention as potential therapeutic agents for PCOS [7]. A number of studies have explored the effects of various plant extracts and natural compounds on PCOS and associated symptoms such as hyperandrogenism, insulin resistance, and menstrual irregularities [8].

*Vitex nigundo* (VN), the five-leaved chaste tree, commonly known as “Nirgundi,” belongs to the Lamiaceae family and is a small deciduous shrub that grows in different tropical and sub-tropical regions of the world, including Asia, Africa, and America [9]. VN has a rich history of being utilized in folk medicine across Bangladesh, India, China, Indo-China, Indonesia, Nepal, Pakistan, Philippines, and Sri Lanka [10]. Every part of this medicinal plant including its leaves, flowers, twigs, roots, and seeds is known to serve a wide range of therapeutic purposes [11]. The pharmacological benefits of Vitex species are widely recognized, with various medicinal properties including antimicrobial, antioxidant anti-inflammatory, and anticancer effects. Scientific research has shown that certain species are particularly effective in treating ailments such as diabetes, as well as improving female health [12].

The seeds of *Vitex nigundo* (VN) are known to contain several active compounds, including flavonoids, iridoids, terpenoids, and essential oils, which are accountable for the plant’s medicinal properties [13]. Recent studies reported the potential therapeutic role of VN in managing the symptoms of PCOS. VN has been shown to reduce serum androgen level, improve insulin sensitivity, and modulate ovarian morphology in animal models of PCOS [14, 15]. These effects may be attributed to the ability of VN to regulate the hypothalamic-pituitary-gonadal axis, decrease oxidative stress, and thereby modulate the expression of genes involved in steroidogenesis.

The bioactive compounds found in the plant’s seeds earlier demonstrated promising results in preclinical studies, indicating their potential for treating PCOS symptoms. However, studies focusing to explore clinical efficacy of *Vitex nigundo* in managing PCOS symptoms are limited. Therefore, further studies are needed to elucidate the potential therapeutic effects of VN in PCOS, exploring its mechanisms of action. To address this gap in knowledge, this study aims to evaluate the effect of VN on reproductive and metabolic parameters with the molecular approach through studying the inflammatory markers as well as apoptotic markers in a rat model of PCOS. These findings will provide the insight into the potential therapeutic effects of *Vitex nigundo* and its mechanisms of action in the treatment of PCOS. Hence, it may be an effective paradigm on the way of overcoming the hazards and low success of conventional therapeutic strategy against PCOS.

## Results

### LC-MS findings of hydro ethanolic extract of *Vitex nigundo*

**Fig. 1:**
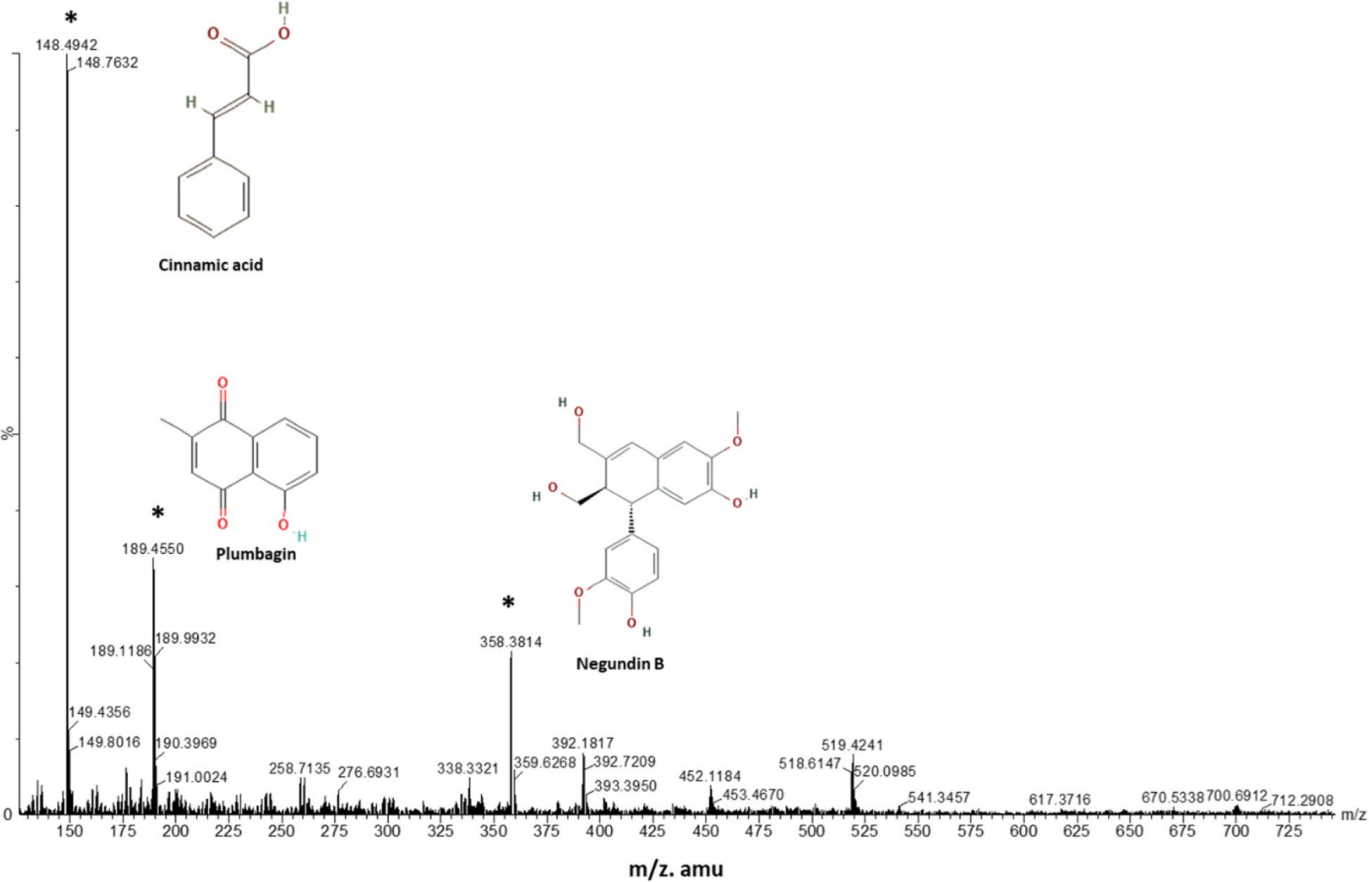
LC-MS analysis of Hydroethanolic extract of *Vitex nigundo*, where data shows 3 different level and different molecular weight peaks; cinnamic acid (148.15 m/z) was found to be most abundant in the extract

LC-MS analysis of the hydroethanolic extract obtained from *Vitex negundo* revealed the presence of three distinct peaks with varying molecular weights. The standard cinnamic acid (C_9_H_8_O_2_) has a known mass of 148.16 (m/z). In this experiment, the extracted components from *Vitex negundo* seeds exhibited the highest peak corresponding to cinnamic acid (Fig. 1), with a mass of 148.49 (m/z), which closely resembled the standard mass of 148.16 (m/z). The second highest peak observed was at 189.45 (m/z), and it is assumed to be plumbagin (C_11_H_8_O_3_) with a molecular weight of 188.18 (m/z). Additionally, another peak was detected at 358.38 (m/z), which is presumed to be negundin B (C_20_H_22_O_6_) with a molecular weight of 358.4 (m/z).

### Bodyweight and Organosomatic Indices

The administration of letrozole for a duration of 21 days resulted in a significant increase in the body weight (*p* < 0.001) of the PCOS group compared to the control rats. However, after treatment with VNE extracts, a significant reduction in body weight (*p* < 0.001) was found in PCOS rats, as presented in Table 1.

**Table 1:** Effect of *Vitex negundo* extract on body weight and organosomatic indices parameters in letrozole induced PCOS rat

Group	Control	PC	LTZ	LTZ + VNE
Changes in body weight (gm %)	116.31 ± 1.81	112.97 ± 2.22 ###	144.48 ± 4.17 ***	117.01 ± 2.47 ###
Ovarian index (gm%)	0.056 ± 0.002	0.057 ± 0.001	0.047 ± 0.083	0.039 ± 0.003
Uterine index (gm%)	0.2263 ± 0.02	0.1834 ± 0.010##	0.0754 ± 0.013 ***	0.1045 ± 0.015

Effect of *Vitex negundo* extract on body weight and organosomatic indices in letrozole induced PCOS rat. Data presented as the Mean ± S.E.M. (n = 6), evaluated by ANOVA followed by the post hoc Tukey's test

*p<0.05, **p<0.01, ***p<0.001 vs. control; #*p*<0.05, ##*p*<0.01, ###*p*<0.001 vs. PCOS group

Both, letrozole (LTZ) and VNE concomitant administration caused a negative effect on ovarian weight although the observed change was insignificant (Table 1). On the other hand, the uterine weight in letrozole induced PCOS group was decreased significantly (*p* < 0.001). However, the supplemented group with VNE found with improved uterine index.

### Metabolic profile

**Fig. 2:**
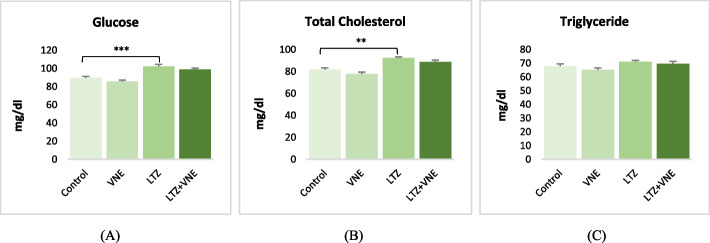
Effects of *Vitex nigundo* on (**A**) Blood glucose (**B**) Total cholesterol (**C**) Triglyceride in rats with letrozole induced PCOS. Data are presented as the Mean ± S.E.M. (*n* = 6), evaluated by ANOVA followed by the post hoc Tukey’s test *P < 0.05, **P < 0.01, ***P < 0.001 vs. control; #*P* < 0.05, ##*P* < 0.01, ###*P* < 0.001 vs. LTZ group

The LTZ-treated groups exhibited a significant increase in fasting blood glucose (*p* < 0.001) and total cholesterol (*p* < 0.01) compared to the control group. Triglyceride level in the LTZ-administered group did not show a significant change. Conversely, following the co-administration of VNE found to show a declining trend in glucose, total cholesterol, and triglyceride level, although no statistical significance was noticed (Fig. 2).

### Activity and expression of Superoxide dismutase (SOD) and Catalase

**Fig. 3:**
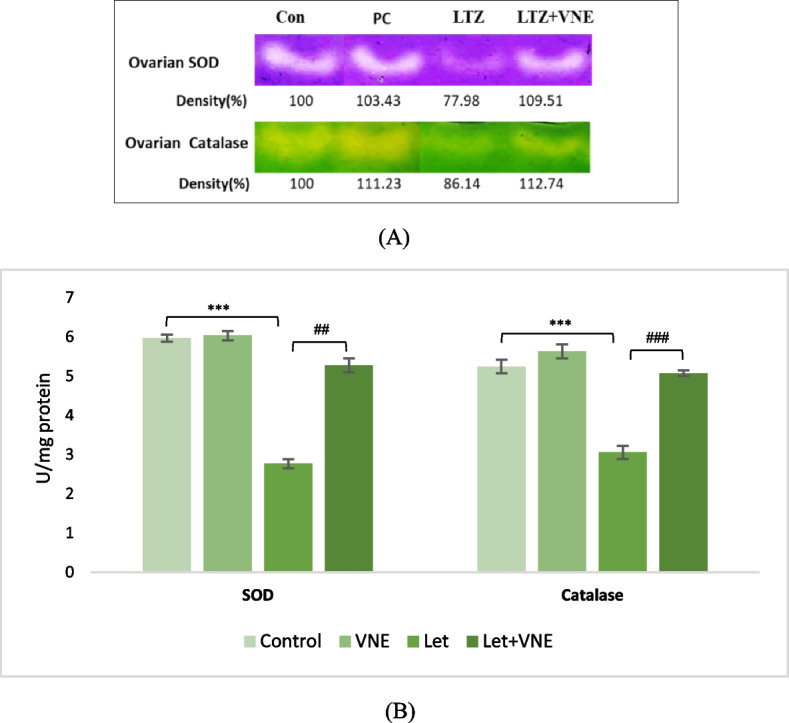
The protective effect of VNE on antioxidative enzyme, superoxide dismutase (SOD) and catalase activities in ovarian tissue was investigated in LTZ-induced PCOS rats. The SOD and catalase activity in ovarian tissue were assessed using agarose gel electrophoresis followed by substrate-specific development of SOD and catalase bands (**A**). The lane distribution on the gel was as follows: Lane 1: control group; Lane 2: VNE group; Lane 3: LTZ-induced PCOS group; Lane 4: LTZ + VNE group. Ovarian tissue extracts containing equal amounts of proteins were subjected to electrophoresis on 12% or 8% native gel and 8% agarose gel, and the substrate-specific development of SOD and catalase bands was performed to evaluate the protective effect of VNE against LTZ-induced changes in SOD and catalase (**A**). Additionally, the spectrophotometric assay of SOD and catalase activity in ovarian tissue was conducted to further investigate the impact of VNE co-administration in LTZ-treated rats (**B**). Data represent as the Mean ± S.E.M. (*n* = 6), evaluated by ANOVA followed by the post hoc Tukey’s test. *P < 0.05, **P < 0.01, ***P < 0.001 vs. control; #*P* < 0.05, ##*P* < 0.01, ###*P* < 0.001 vs. LTZ group

Spectrophotometric and electrozymographic analysis were employed to evaluate the activities and expression of antioxidant enzymes, respectively superoxide dismutase and catalase, in ovarian tissue. The findings depicted in Fig. 3A clearly demonstrated a meaningful suppression in the expression of MnSOD and catalase in PCOS rats when compared to the control group. This declination was visually evident from the faint bands observed in the ovarian tissue analysis. Conversely, the PCOS group co-administered with VNE exhibited a noteworthy restoration in its protein expression status. This restoration was substantiated by the appearance of more intense bands of these antioxidant enzymes of VNE co-administered group. Similar fashion of above result was reviewed when enzyme kinetics of above antioxidants were considered (Fig. 3B).

### Serum hormones, steroidogenic enzymes and ER-α

The result showed in Table 2, depicts a minimal increase in estradiol though insignificant in the LTZ induced PCOS group but VNE treatment in LTZ induced rats consequentially weaken the estradiol signaling. Conversely, testosterone level in PCOS rats was significantly elevated, approximately 2-fold higher compared to the control group, indicating a pronounced increase. However, VNE in LTZ induced rats with PCOS exhibited its antiandrogenic properties, leading to a substantial decrease in testosterone level (Table 2). Furthermore, the PCOS group displayed a significantly dropped in progesterone release, approximately half of the control group (*p* < 0.001). On the contrary VNE co-administration resulted in a remarkable surge of progesterone (*p* < 0.001).

**Table 2:** Effect of *Vitex negundo* extract on Serum hormones, steroidogenic enzymes and Estrogen receptor in letrozole induced PCOS rat

Parameters	Control	VNE	LTZ	LTZ + VNE
Estradiol (pg/ml)	587.65 ± 16.16	335.86 ± 19.34 ###	646.02 ± 34.93	366.00 ± 6.98 ###
Progesterone (ng/ml)	104.45 ± 4.84	116.84 ± 3.15 ###	65.07 ± 3.75 ***	117.34 ± 3.16 ###
Testosterone (ng/ml)	1.12 ± 0.215	1.05 ± 0.135 #	2.58 ± 0.522 *	1.16 ± 0.179 #
3β-HSD (ng/ml)	284.21 ± 13.40	280.94 ± 10.36 ###	396.08 ± 7.53 ***	375.86 ± 9.58
Aromatase (pg/ml)	1038.87 ± 34.55	1024.07 ± 33.56 ###	1522.62 ± 36.40 ***	1287.15 ± 48.06
ER-α (ng/ml)	3.731 ± 0.158	3.442 ± 0.170 ###	4.324 ± 0.159 *	3.125 ± 0.035 ###

Effects of *Vitex nigundo* on serum hormones (estradiol, progesterone and testosterone), steroidogenic enzymes (3β-HSD and aromatase), ER-α in rats with letrozole induced PCOS. Data presented as the Mean ± S.E.M. (*n* = 6), evaluated by ANOVA followed by the post hoc Tukey's test

*p<0.05, **p<0.01, ***p<0.001 vs. control; #*p*<0.05, ##*p*<0.01, ###*p*<0.001 vs. PCOS group. The inter and intra assay variation (CV) was <10 % for all parameters

Additionally, the results presented in Table 2, indicated a significantly higher concentration of steroidogenic enzyme 3β-HSD and aromatase in PCOS (*p* < 0.001). Concomitant administration of VNE along with LTZ did not showed in any significant changes when compared with PCOS group, though there was very minimal changes of aromatase and 3β-HSD were observed.

Furthermore, the expression of ER-α in ovarian tissue was found to enhance in the PCOS group. However, following the co-treatment of VNE in LTZ fed rats with PCOS exhibited a meaningful ER-α repression (Table 2).

**Fig. 4:**
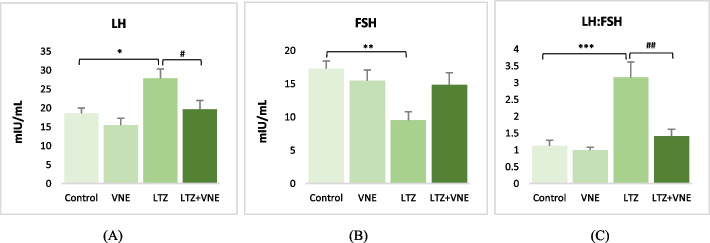
Effects of *Vitex nigundo* on (**A**) LH (**B**) FSH (**C**) LH:FSH ratio in rats with letrozole induced PCOS. Data are presented as the Mean ± S.E.M. (*n* = 6), evaluated by ANOVA followed by the post hoc Tukey’s test. *P < 0.05, **P < 0.01, ***P < 0.001 vs. control; #*P* < 0.05, ##*P* < 0.01, ###*P* < 0.001 vs. LTZ group. The inter and intra assay variation (CV) was < 10% for all parameters

A LH upsurge was significantly noted in the LTZ-induced PCOS group. However, the concurrent administration of VNE had an inhibitory effect on LH level, leading to 1.5-fold reduction approximately in a significant manner. In contrast, LTZ administration markedly reduced FSH levels by almost half compared to the control group. In contrast, cotreatment with VNE did not appear to influence FSH levels relative to the PCOS group. The LTZ-treated group exhibited a significant threefold increase in the LH:FSH ratio, indicating an imbalance in gonadotropins, as depicted in Fig. 4. However, VNE treatment seemed to correct this ratio (1.4), closely approaching the level (1.1) observed in the control group. This suggests the potential of VNE in restoring the balance of these gonadotropins.

### Analysis of ovarian inflammatory status and associated factors

**Fig. 5:**
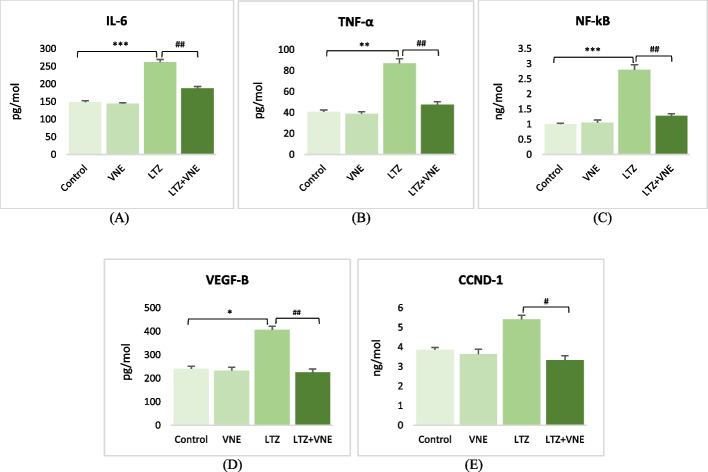
Effects of *Vitex nigundo* on (**A**) IL-6 (**B**) TNFα (**C**) NF-ĸB (**D**) VEGF-B (**E**) CCND-1in rats with letrozole induced PCOS. Data are presented as the Mean ± S.E.M. (*n* = 6), evaluated by ANOVA followed by the post hoc Tukey’s test. *P < 0.05, **P < 0.01, ***P < 0.001 vs. control; #*P* < 0.05, ##*P* < 0.01, ###*P* < 0.001 vs. LTZ group. The inter and intra assay variation (CV) was < 10% for all parameters

The impact of VNE on the inflammatory status of ovarian tissue and associated factors was established in letrozole-induced PCOS rats. As shown in Fig. 5, the level of proinflammatory cytokines, including IL-6 and TNFα, along with NF-ĸB, were significantly elevated approximately 2 to 3-folds in the polycystic ovarian tissues. However, VNE administration explored a noteworthy effect (*p* < 0.01) in reducing these markers of ovarian inflammation.

A marked elevation (*p* < 0.01) of VEGF-B level was noted in PCOS rats while the minimal elevation of CCND-1. It was insignificant in this group. Nevertheless, VNE treatment in PCOS rats exhibited a consequential reduction of these two biomarkers (Fig. 5D and E).

### mRNA expression of proinflammatory markers

**Fig. 6:**
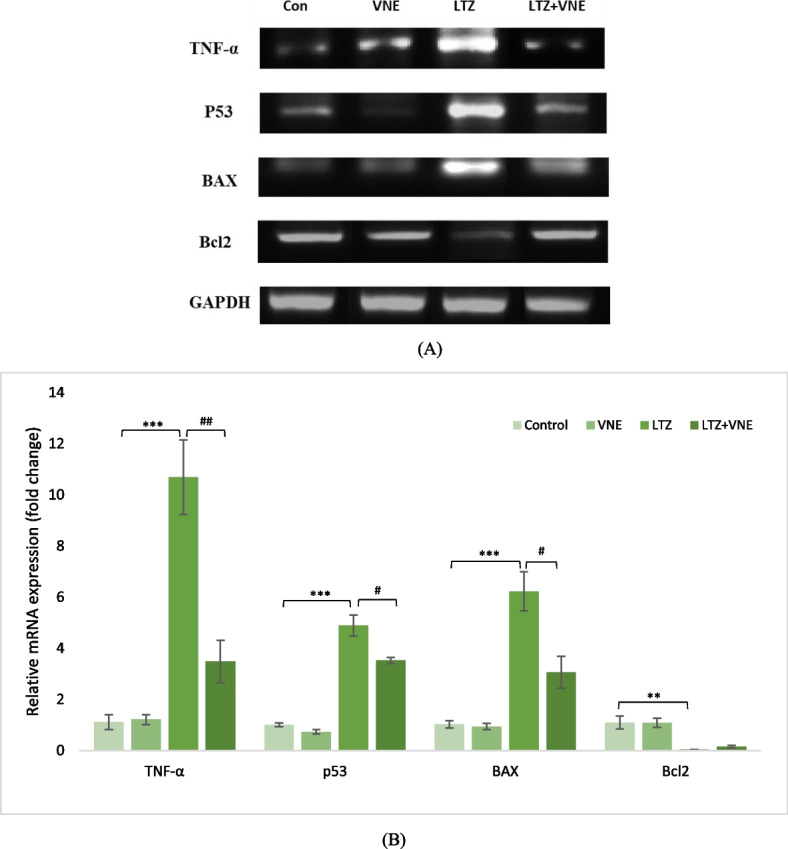
** A** The impact of VNE on apoptotic and pro-inflammatory changes in mRNA expression activity was investigated in LTZ-induced PCOS rats. The genes TNF-α, p53, BAX and Bcl-2 were assessed using PCR analysis, with GAPDH serving as the reference gene. This study aimed to examine the effects of VNE on the expression level of these mRNA associated with apoptosis and inflammation in the PCOS model induced by LTZ. **B** The impact of VNE on apoptotic and pro-inflammatory changes in mRNA expression activity was investigated in LTZ-induced PCOS rats. The genes TNF-α, p53, BAX and Bcl-2 were assessed using Real time PCR analysis, with GAPDH serving as the reference gene. Data presented as the Mean ± S.E.M. (*n*  = 3), evaluated by ANOVA followed by the post hoc Tukey’s test. * *P*  < 0.05, ** *P*  < 0.01, *** *P*  < 0.001 vs. control; # *P*  < 0.05, ## *P*  < 0.01, ### *P*  < 0.001 vs. LTZ group

Establishing the impact of VNE on mRNA expression of proinflammatory markers in ovarian tissue and its potential effect against PCOS, the expressions of TNF-α, p53, BAX and Bcl-2 in the ovarian tissue were assessed at the RNA transcript level. Semiquantitative RT- PCR and qRT-PCR were conducted to analyze the mRNA expressions. The results indicated an upregulation of ovarian TNF-α, BAX and p53 in the PCOS group following LTZ administration compared to the control ovaries (Fig. 6A). The results of qRT-PCR analysis of relative mRNA displayed in the Fig. 6B further supported the upregulation of TNF-α (10.69-fold), p53 (4.89-fold), BAX (6.23-fold), and downregulation of Bcl2 (0.041-fold) in PCOS rats (Fig. 6B). However, following the administration of VNE, these aberrations of mRNA expression were reversed, as observed in Fig. 6A and B.

### Ovarian histopathology

To assess the potential therapeutic effects of VNE on PCOS, we investigated its impact on ovarian morphology in rats. Letrozole-treated rats exhibited polycystic ovarian morphology, characterized by fewer normal follicles at various developmental stages. The PCOS group showed a decrease in primordial, secondary, tertiary follicles, and an increase in cystic follicles, confirming successful model establishment. Moreover, the letrozole-treated group displayed disorganized ovarian tissue, marked by numerous subcapsular cystic follicles lacking oocytes and corona radiata. Additionally, the PCOS group exhibited an increased thickness of the theca and tunica albuginea layers compared to the control group. Significant differences were noted among the groups in terms of the number of multilaminar follicles, thickness of the granulosa and zona pellucida layers.

**Fig. 7:**
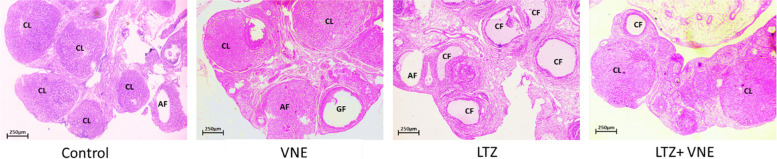
Effect of *Vitex negundo* extracts on ovarian morphology in different groups of rats: **A** Control **B** VNE **C** LTZ **D** LTZ + VNE group are presented at 40X magnification. Graafian follicle (GF), atretic follicle (AF), cystic follicles (CF), corpus luteum (CL) are indicated by super script

Conversely, the concurrent application of VNE in PCOS rats resulted in a reduction in cyst n umbers, an increased presence of corpus luteum and a significant decrease in the thickness of the theca and tunica albuginea layers, suggesting a restoration of normal folliculogenesis and ovulation. The improved follicular morphology in PCOS rats co-treated with VNE exhibited a substantial reduction in pathological damage to the ovaries, indicating positive effects on overall ovarian histoarchitecture, as depicted in Fig. 7.

### Immunohistochemical staining of androgen receptors

**Fig. 8:**
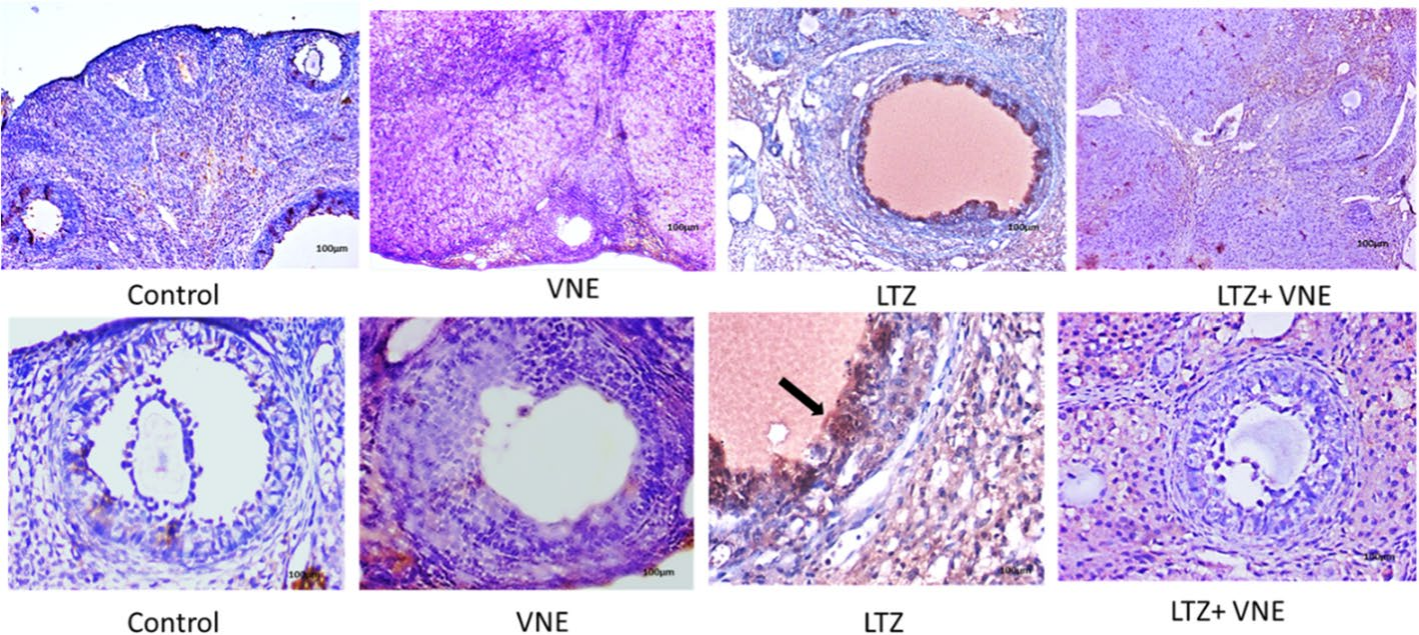
Microscopic images show effect of VNE on the NR3C4 expression in the ovarian tissues in a polycystic ovary syndrome rat model. Immunohistochemical staining of NR3C4 in different experimental groups: **A** Control **B** Positive control **C** LTZ **D** LTZ + VNE group. Brown staining of the cells indicates the expression of AR (100 X & 400X)

Immunolocalization and expression of NR3C4 in ovarian sections of POCS rats are illustrated in Fig. 8. Higher NR3C4 expression was observed mainly surrounding the cystic follicle of ovaries in PCOS group. However, a decrease in NR3C4 expression in the ovarian cells in VNE co-treated group was noticed.

### Illustration of cinnamic acid, plumbagin and negundin b

**Fig. 9:**
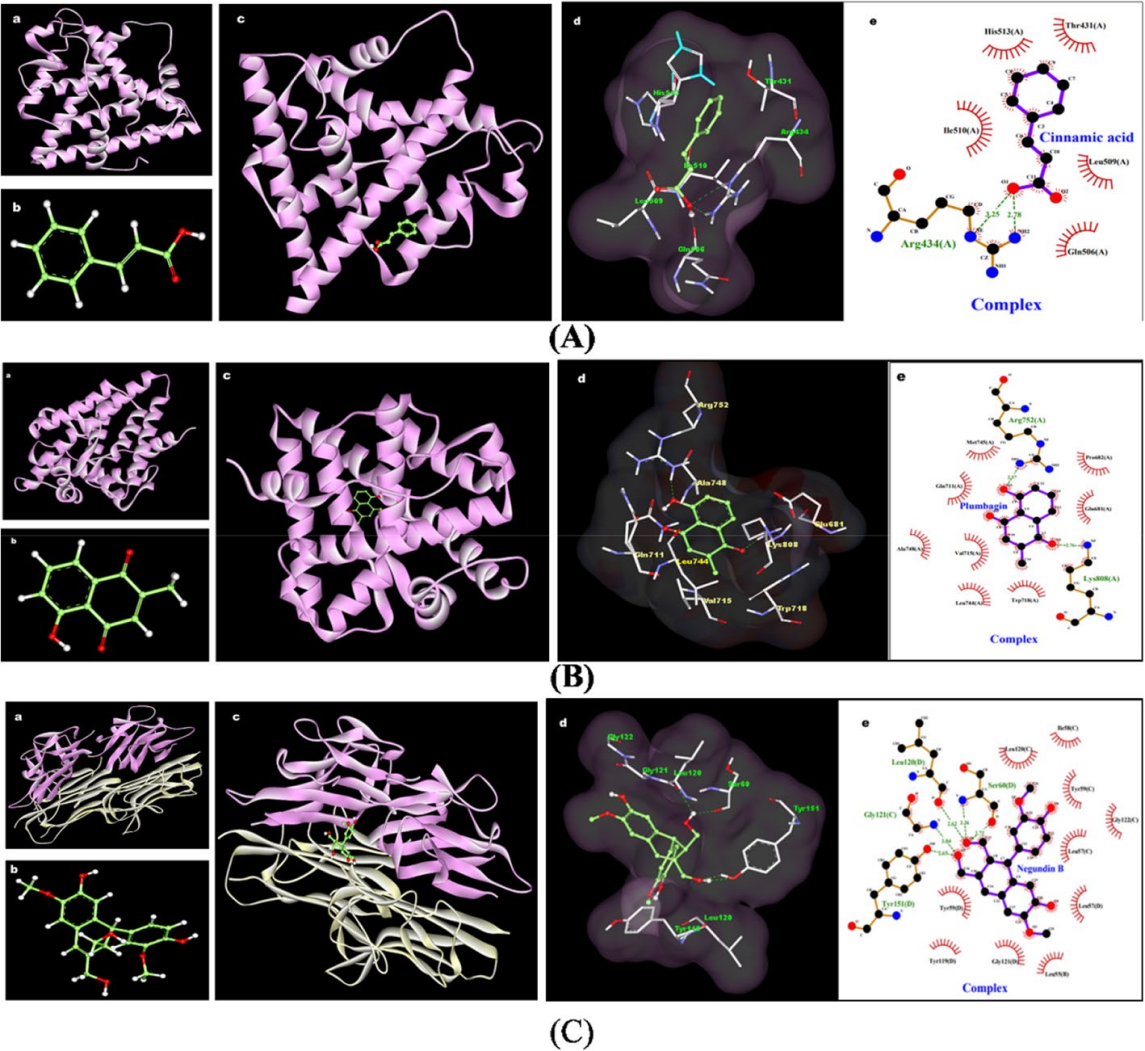
**A** Molecular interaction of cinnamic acid with ER-α. (a) 3D structure of human Estradiol receptor alpha (PDB ID- 3ERT). (b) 3D ball and stick structure of cinnamic acid (PubChem ID- 444539). (c) 3D molecular docking image of cinnamic acid with Estradiol receptor alpha (Biovia Discovery Studio Visualizer). (d) 3D molecular interaction image of cinnamic acid shows involvement of Thr431, Arg434, Gln506, Leu509, Ile510 and His513 (Biovia Discovery Studio Visualizer). (e) 2D molecular interaction image of cinnamic acid binding sites along with the bond distance. Green dashed line defines the hydrogen bond interaction whereas red lines hydrophobic interactions. (Ligplot+ v.2.2.4). **B **Molecular interaction of plumbagin with NR3C4. (a) Crystal 3D structure of human androgen receptor ligand binding domain in complex with testosterone retrieved from protein data bank. PDB ID: 2AM9 (b) 3D ball and stick structure of plumbagin. PubChem ID: 10205. (c) 3d Molecular docking image of plumbagin interacting with the ligand binding domain of androgen receptor. (d) 3d representation the binding pocket of plumbagin in the ligand binding domain of androgen receptor (Biovia discovery studio visualizer). (e) 2D representation of hydrogen bond interaction (green dashed line) of plumbagin along with bond-distance and hydrophobic interactions (arcs). (Ligplot+ v.2.2.4). **C **Molecular interaction of negundin b with TNFα.(a) 3D structure of human TNFα protein (PDB ID- 2AZ5). (b) 3D ball and stick structure of Negundin B (PubChem ID- 10473569). (c) 3D molecular docking image of Negundin B with tumor necrosis factor alpha. (Biovia Discovery Studio Visualizer). (d) 3D view of close molecular interaction image of negundin b with interactive amino acids. (Biovia Discovery Studio Visualizer). e. 2D molecular interaction image of negundin b involves Leu55, Leu57, Ile58, Tyr59, Ser60, Tyr119 Leu120, Gly121 and Tyr151 along with the bond distance. Green dashed line defines the hydrogen bond interaction whereas red lines hydrophobic interactions. (Ligplot+ v.2.2.4)

The possible interactions of cinnamic acid with the c-terminal ligand binding domain of estradiol receptor alpha (ESR-α) involves Arg434 (hydrogen bond interaction) Thr431, Gln506, Leu509, Ile510 and His513 (hydrophobic interactions) (Fig. 9A). Plumbagin displayed the interactions with LBD of NR3C4 and showed a possible interaction with Arg752, Lys808 (hydrogen bond interaction) Glu681, Pro682, Trp718, Val715, Ala784 and Gln711 (hydrophobic interaction) (Fig. 9B). On the other hand, the catalytic site of negundin b showed a possible interaction with Ser60, Leu120, Gly121, Tyr151 (hydrogen bond interaction), Leu55, Leu57, Ile58, Tyr59 and Tyr119 (hydrophobic interaction) of TNF-α dimer (Fig. 9C). Interaction of negundin b with these crucial amino acids may exhibit a potential inhibitory effect on TNF-α-mediated inflammatory pathway during PCOS. Binding energy of cinnamic acid with ESR- α, plumbagin with NR3C4 and negundin b with TNF-α are − 5.34 kcal/mol, -7.26 Kcal/mol and − 6.28 kcal/mol respectively. Root Mean Square Deviation (RMSD) value = 0.00.

## Discussion

PCOS, a heterogeneous condition associated with chronic inflammation, oxidative stress, and metabolic disturbances deleteriously affects the quality of life and long-term health of affected individuals with the condition [5]. Unfortunately, no satisfactory treatment has been identified for PCOS thus so far. This study aimed to investigate the therapeutic effects of the ethanolic extract of *Vitex nigundo* seed (VNE) on letrozole induced PCOS. Letrozole, a targeted non-steroidal aromatase inhibitor, suppresses aromatase, leading to elevated testosterone levels and hyperandrogenism, replicating various PCOS phenotypes. Despite letrozole administration, there was a surprising increase in ovarian aromatase. Since only ovarian tissue was quantified and aromatase activity wasn’t directly measured, it’s unclear why aromatase expression remains significantly higher despite the use of an aromatase inhibitor. Though the concentration of aromatase is higher, at that extent the activity of aromatase may not sufficiently be achieved which reflected from the very negligible increase in circulating estradiol level. It is plausible that, this might stem from feedback associated with competitive enzyme inhibition by letrozole. Our findings also reveal a significant increase in testosterone, indicating an impaired aromatization of testosterone to estrogen. The heightened substrate concentration might partially overcome competitive inhibition by letrozole. A subtle, statistically non-significant, overproduction of estrogen might result from this intricate phenomenon which was reported previously in recent study [16]. In this study, letrozole is hypothesized to hinder aromatase activity, inhibiting androgen conversion, resulting in clinically observed elevated testosterone, increased LH, and decreased FSH levels, causing a reproductive hormonal imbalance and multiple cysts development as a result of ovulatory disparities.

Our findings demonstrated the positive impact of VNE on alleviating PCOS symptoms attributed to its potent anti-inflammatory and antioxidant properties along with its regulatory role on reproductive hormones. These results highlight the potential of VNE as a treatment option for PCOS related reproductive and metabolic disorders, addressing the unmet need for effective PCOS management.

Almost 50% of women afflicted with PCOS exhibit overweight or obesity [17]. Excess body weight aggravates insulin resistance followed by metabolic aberration, hyperandrogenism, inflammation and infertility in individuals with PCOS [18]. In current study, we observed the anabolic effect of letrozole on rats with significantly increased relative body weight. However, the hydroethanolic extract of *Vitex nigundo* seed found to be effective in weight reduction (Table 1). Previous studies are also showing the evidence of similar fashion of weight reducing potential of *Vitex nigundo* seed [14, 15]. Obesity and body fat percentage have a direct effect on serum estrogen release as a result of increased peripheral aromatizati1on in adipose tissue. This directly impact the LH:FSH ratio [19]. Administration of VNE in the present investigation found with correction of LH:FSH ratio (Fig. 4C). This might be attributed to the direct inhibition of LH production pertinent to the action of cinnamic acid present in *Vitex negundo* seed extract, which was documented earlier for interaction with estrogen receptor (ER) in the hypothalamus [20]. Molecular docking analysis in the present study further supports the ability of cinnamic acid in modulating ER signalling through limiting the overexpression of ER-α in the ovary (Fig. 9B). Since, the C-terminus part of ER-α contains a ligand-mediated transactivation domain, binding of cinnamic acid to ligand binding domain may limit the overexpression of ER-α followed by altered ER signalling in PCOS. Thus, reestablishing the balance in hypothalamus–pituitary–ovary (HPO) axis leading to reduction in LH followed by the correction of steroidogenic enzymes aromatase as well as 3β-HSD. Cinnamic acid also been found to inhibit the functional activity of 17β-HSD as well as 3β-HSD leading to decreased production of testosterone [21, 22]. Modulating the HPO axis along with regulating steroidogenesis and gonadotropin release, VNE exerts a profound effect on restoring the homeostasis of ovarian hormones, specifically estrogen and progesterone. This restorative mechanism is intricately associated with the elevation of progesterone levels (Table 2), which in turn correlates with augmented numbers of corpus luteum (Fig. 7), indicative of successful ovulation and improved fertility. While, LTZ-induced PCOS rats exhibited increased numbers of follicular cysts with thickened theca cell layer and decreased presence of follicles at different stages (Fig. 7). This morphological change is associated with altered LH:FSH ratio that triggers the proliferation of ovarian theca cells, leading to heightened androgen production, by upregulated steroidogenic enzymes ultimately resulting in hyperandrogenism in PCOS [23]. Diverse patterns of ER expression in various ovarian cells control follicular growth and development. In PCOS, altered expression of ERα and ERβ is linked to abnormal follicular growth and ovulatory dysfunction [24]. Estrogen stimulates thecal androgen production [25]. In PCOS, increased ERα in ovarian thecal cells indicates heightened responsiveness to estrogen, leading to overproduction of androgens. Our histological observation of increased theca cell thickness supports excess androgen production and aligns with elevated ovarian ER-α expression.

The result of the current study depicted in the Fig. 8 displayed an increased immune-expression of NR3C4 or AR protein within the ovarian cells of LTZ treated rats and is in agreement with the observation of others investigations in this time [26, 27]. Upregulation of NR3C4 might be due to the accumulation of endogenous testosterone resulting in impaired folliculogenesis and ovulatory dysfunction [28]. On the other hand, supplementation of VNE showed an antiandrogenic action by suppressing expression of ovarian NR3C4 followed by suppressed serum testosterone release (Table 2). This action of VNE might be ascribed to the possible interaction of plumbagin with NR3C4 indicated by molecular docking analysis in the present study (Fig. 9B). Our study revealed that plumbagin formed hydrogen bonds with Arg752 and Lys808, while also engaging in hydrophobic interactions with Gln711 in the ligand binding domain (LBD). Earlier research highlighted Arg752 and Gln711 as pivotal amino acids for testosterone binding to the LBD of androgen receptor [29]. Analyzing crystal structures of human androgen receptor ligand-binding domains complexed with different agonists has yielded valuable insights into the determinants influencing binding affinity. The interaction between plumbagin and these crucial amino acids (Arg752 and Gln711) likely underpins the observed inhibitory impact on androgen signalling. This interaction could serve as a potential mechanism through which VNE exerts its effects on androgen signalling pathways.

Elevated testosterone levels, acting through androgen receptors in pancreatic cells and hepatocytes, result in hyperinsulinemia and compromised metabolic functions [30]. This sequence of events leads to insulin resistance and the consequent progression of type 2 diabetes mellitus in individuals affected with PCOS. The current study showed that administration of VNE resulted in notable reduction in plasma glucose level in LTZ induced PCOS rats (Fig. 2A). Earlier study also established a hypoglycemic effect of VN in PCOS rats [14]. The hypoglycemic effect could be due to the presence of cinnamic acid and plumbagin that directly influences glucose digestion, absorption and metabolism including increased glucose uptake by restoring GLUT4 translocation to plasma membranes [31, 32]. Assessment of lipid profile in this study revealed hypocholesterolemic effect of VNE. However, no significant change found in triglyceride level either in LTZ treated group or in VNE treated group. The positive control treated only with the VNE showed a hypolipidemic effect which was statistical significance as compared to treated group (Fig. 2B & C). The positive effect on regulation of plasma lipids profile might be due to antioxidant property of active components of VNE [12].

Oxidative stress is another prominent feature of PCOS and is associated with insulin resistance, disrupted steroidogenesis, increased lipid peroxidation, and activation of inflammatory cytokines, leading to impaired reproductive function and metabolism [33]. Oxidative stress hinders the production and release of SHBG by reducing the activity of HNF-4α, which could potentially play a significant role in the development of hyperandrogenism in PCOS [34]. Several studies highlight substantial mitochondrial dysfunction and heightened oxidative stress in PCOS, influencing both reproductive and transgenerational outcomes [35]. In the present study, we examined the expression of MnSOD, localized in mitochondria, emphasizing its direct relevance in addressing PCOS-associated mitochondrial issues. In line with previous reports on LTZ-induced PCOS, the PCOS group in this study demonstrated decreased activity of antioxidant enzymes [36]. However, treatment with VNE successfully protected the ovarian tissue from oxidative stress induced by PCOS (Fig. 3A and B). This might be associated with its active components that could counteract oxidative damage, leading to correction of antioxidant enzyme status in ovarian tissue. This antioxidant action may protect ovarian tissue from oxidative stress thereby preserving follicular health, and improve reproductive outcomes in PCOS.

In the realm of PCOS, oxidative-inflammatory cascades play a prominent factor intricately associated with the pathogenesis and detrimental consequences of the condition [37]. The pivotal role of proinflammatory cytokines has been well-established in regulating ovarian steroidogenesis, follicular maturation, and ovulation, as well as contributing to the development of insulin resistance and hyperandrogenism in PCOS [37]. The interplay between TNF-alpha, IL-6, and NF-ĸB forms a complex signalling pathway that regulates inflammation and immune responses. The current study observed a notably upregulated expression of TNF-α, IL-6 as well as NF-κB in the PCOS group, which was accompanied with metabolic disturbances and hyperandrogenism (Figs. 5 and 6). The efficacy of VNE as an anti-inflammatory agent, as demonstrated earlier [38], was further corroborated by our findings. Present study revealed a significantly repressed expression of TNF-α, IL-6 along with NF-κB in ovarian tissue following VNE supplementation (Figs. 5 and 6). Notably, plumbagin one of the major components found in the seed of VNE (Fig. 1), has been reported to directly inhibiting NF-ĸB activation, subsequently leading to the sequential suppression of TNF-α and other inflammatory cytokines [39]. In addition, Negundin-B, another active compound found in VNE, has been associated with the inhibition of lipoxygenase, an oxidative enzyme [40]. A study found the potential of lipoxygenase inhibitors to reduce TNF-α effectively followed by attenuation of inflammation [41]. Furthermore, present study demonstrates the ability of negundin B to bind with the TNFα-dimer interface that hinders the interaction of TNFα with its receptors (Fig. 9C) which in turn affects the downstream signalling pathways of inflammation.

The interplay between oxidative stress, inflammation, and apoptosis contributes to ovulatory dysfunction in PCOS. Oxidative stress-induced inflammation upregulates the expression of pro-inflammatory cytokines such as TNF-α, promoting apoptosis [42]. Moreover, androgens exhibit proinflammatory properties by the activation of nuclear factor of T-cells (NFAT5), which subsequently triggers the upregulation of TNF-ɑ and IL-1β followed by apoptosis of granulosa cell [43, 44]. Furthermore, prior research indicated the involvement of androgens in facilitating BAX-mediated apoptosis and also negatively impact on the expression of Bcl-2 [45, 46]. Subsequently dysregulated expression of apoptosis-related proteins, including increased BAX and p53, and suppressed Bcl-2 further disrupts the delicate balance between cell survival and death, ultimately leading to impaired ovulation in PCOS [47]. This association is reaffirmed by our present study, which demonstrates a concurrence between elevated androgen levels, heightened NR3C4 expression in a PCOS model, and upregulated expression of p53 and BAX along with downregulated expression of anti-apoptotic Bcl-2. However, VNE administration successfully restored these markers in ovarian tissue, indicating its antiapoptotic role in PCOS (Fig. 6A & B).

VEGF is produced by follicular granulosa and ovarian theca cells in response to gonadotropin stimulation [48]. Additionally, evidence indicates that estrogen primarily through ERα regulates VEGF transcription [49]. Individuals with PCOS exhibit higher concentration of VEGF-B in serum and ovarian follicular fluid, a prominent angiogenic factor, which is positively correlated with insulin insensitivity and inflammation [50]. Elevated VEGF may be the indicator of early changes arising during the progression of PCOS towards hyperplasia and cancer [51]. Present study also evident a similar pattern of VEGF-B level in PCOS group which was brought down following the administration of VNE (Fig. 5). In PCOS, elevated CCND-1 levels, a key regulator of the cell cycle, are associated with inflammation. The concurrent upregulation of ERα expression, along with inflammation, plays a pivotal role in estrogen-induced proliferation by initiating CCND-1 gene transcription and overexpression [52]. This process contributes to abnormal cell proliferation and disturbances in ovarian follicular development, defining the features of PCOS [53]. Present study demonstrates VNE supplementation to alleviate the elevated signalling of CCND-1 in PCOS (Fig. 5). These reduction in VEGF-B and CCND-1 suggests that VNE may exert modulatory effects on key processes such as inflammation-associated angiogenesis and folliculogenesis, potentially through the regulation of cell cycle progression.

## Conclusions

VNE presents a promising avenue for addressing PCOS given its multi-dimensional effects rooted in strong antiandrogenic, anti-inflammatory, and antioxidant properties. Key compounds like cinnamic acid, plumbagine, and nigundin B are notably linked to these attributes. VNE impacts weight reduction, improves metabolic profiles, and mitigates hyperandrogenism by targeting LH:FSH ratio and regulating NR3C4 and ER-α. It also protects ovarian tissue from oxidative damage through the correction of SOD and catalase. Additionally, the ability of VNE to regulate the expression of TNF-α, BAX, Bcl2, p53, and modulate VEGF and CCND-1 levels suggests a role in countering inflammation and angiogenesis. In essence, this study suggests VNE as an efficient herbal remedy for combating PCOS, addressing inflammation, oxidative stress, and reproductive hormonal imbalances. However, further studies are needed to confirm active components, and a well-defined mechanistic view requires in-depth analysis of steroidogenic enzyme activity and expression of different associated proteins.

## Methodology

### Chemicals and reagents

The study utilized analytical grade chemicals and reagents sourced from reputable suppliers, including Hi Media (India) and Merck (India) and all consumables were of standard quality.

### Procurement and validation of the plant material

The seeds of *Vitex negundo* were purchased locally (Yuvika, India, batch No. YUVI0357, Licence No. 13,317,002,000,140, FDA approved) and the seeds sample of *Vitex negundo* was verified by Dr. Dulal Kumar De, Professor, Taxonomist, Department of Botany, Midnapore College, Midnapore, West Bengal, India.

### Preparation of Hydro-ethanolic extracts of *Vitex negundo *seeds

The seeds were dried for 48 h at 40℃ in an incubator before being ground with an electrical grinder. 100 g of seed powder were combined with 100 ml of a solvent made up of 70% ethanol and 30% distilled water, and the mixture was shaken occasionally for three days. The liquid, extract was filtered and placed in a beaker after three days. The liquid dark brown extract was dried up and preserved as a dry powder. This dried powder was weighed out and properly dissolved in distilled water prior to treating the rat. The extracts were stored until further usage in sterile bottles in a refrigerator.

### LC-MS analysis of hydro-ethanolic extract of *Vitex nigundo*

LC/MS analyses of plant extracts were conducted following the method by Wojakowska et al., 2013 [54], using a Waters Acquity UPLC system connected to a Bruker Daltonics micrOTOF-Q mass spectrometer, along with the Tri-Versa Nano-Mate system. Chromatographic separation employed Poroshell 120 EC-C18 columns, with an injection volume of 5 ml and a flow rate of 0.6 ml/min. Solvent mixtures A (99.5% H_2_O/0.5% formic acid) and B (99.5% acetonitrile/0.5% formic acid) were used, and elution steps followed a specific gradient pattern. The micrOTOF-Q operated in positive and negative ion modes, with optimized settings for ionization and fragmentation. Flavonoid conjugates were detected by comparing molecular masses and fragmentation patterns. The TriVersa NanoMate system was introduced to facilitated fraction collection with improved separation efficiency.

### Dose selection and care of experimental animals

The administration of *Vitex negundo* extract has been investigated in previous studies using dosages ranging from 200 to 400 mg/kg body weight [14, 15]. In line with these findings, we selected a moderate dose of 250 mg/kg body weight for the current investigation. In this study, 24 female albino rats (100 ± 10 g body weight, 6–8 weeks old) were acclimated for 8 days at a temperature of 25 °C and a relative humidity of 50–70% in the animal house. Rats were kept in cages made of polycarbonate and were provided access to food and water ad libitum. The animals were procured from licensed animal suppliers. The experimental protocols followed the Committee for the Control and Supervision of Experiments on Animals CCSEA (former CPCSEA) guidelines and were approved by the institutional ethics committee (VU/IAEC/CPCSEA/17/7/2022).

### Animal treatment

Six groups of animals were included in the study, with each group comprising six animals (*n* = 6). With the exception of Group I, all animals were administered 1 mg/kg letrozole dissolved in 1% CMC (2 ml/kg) once daily for 21 days. The induction of PCOS was confirmed by evaluating vaginal smears collected daily and examined microscopically using Giemsa stain. Group I (CON) received carboxymethyl cellulose (CMC) as vehicle and was fed a standard diet of rat chow and water ad libitum throughout the experiment. Group II (VNE) received only VNE (250 mg/kg body weight/day), while Group III (LTZ) received 1 mg/kg body weight of letrozole, dissolved in CMC. Group IV (LTZ with VNE) received a combination of 250 mg/kg VNE and 1 mg/kg letrozole orally once a day throughout the experiment.

### Organosomatic indices

The body weight of all animals was documented at the beginning and at weekly interval throughout the experiment. The weight of relevant organs was also recorded after the sacrifice of the experimental rats.

### Serum metabolic parameters

After collecting the blood samples from rats, centrifugation was performed at 2,500 rpm for 5 min to separate serum. To measure the fasting glucose, total cholesterol (TC), triglyceride (TG) level in the serum, the recommended methods by the manufacturer’s guidelines (ENZOPAK) were employed.

### Spectrophotometric assays of antioxidant enzymes (SOD & catalase)

Ovarian tissues were homogenized in Tris-HCl buffer (pH 7.4), and the resulting supernatant was mixed with thio barbituric acid (TBA), 7.5 mmol/L nicotinamide adenine dinucleotide phosphate-reduced, nicotinamide adenine dinucleotide phosphate-reduced-MnCl2. The SOD (superoxide dismutase) activity was then measured at a wavelength of 340 nm [55].

To assess catalase activity, a heated dichromate in acetic acid solution was added in presence of H_2_O_2_. This mixture underwent a transformation into perchromic acid and finally to chromic acetate. The formation of chromic acetate was measured at a wavelength of 570 nm [56].

### Electrozymographic analysis of SOD and Catalase

Ovarian tissue extract was made ready by mixing the tissue with ice-cold phosphate buffer saline (PBS) at a concentration of 20% (w/v), followed by obtaining the supernatant. To detect SOD (MnSOD), a 12% native polyacrylamide gel electrophoresis technique was utilized. The gels were then treated with a solution containing tetramethyl ethylene diamine, nitro blue tetrazolium, and riboflavin, and placed in the dark to visualize colourless MnSOD bands under fluorescent light [56]. To detect the existence of catalase and peroxidase, an 8% native polyacrylamide gel electrophoresis method was employed. The gels were immersed in a solution containing 0.003% H_2_O_2_ and then incubated with a mixture of 2% potassium ferricyanide and ferric chloride. The catalase bands appeared colourless against a blue-green background [56].

### ELISA of serum reproductive hormones ER-α and steroidogenic enzyme (3βHSD)

ELISA kits were used to measure the serum level of estradiol (ELK Biotechnology; Cat:ELK8714), progesterone (ELK Biotechnology; Cat: ELK8385), testosterone (ELK Biotechnology; Cat: ELK8314), luteinizing hormone (LH) (CUSABIO; Catalog Number: CSB E12654r), follicle stimulating hormone (FSH) (CUSABIO; Catalog Number. CSB-E06869r) and tissue level of Estradiol receptor- α (ABclonal; cat: RK07561), 3-beta-hydroxysteroid dehydrogenase (3β-HSD) (FineTest; cat: ER0665) and aromatase (ABclonal; cat: RK04428). The manufacturers’ recommended procedures were followed, and the spectrophotometric reading was taken at 450 nm. Progesterone was assessed using competitive ELISA method where as other parameters were analysed using sandwich ELISA.

### Assessment of ovarian tissue level of IL-6, TNFα, NF-ĸB, VEGF-B, CCND-1through ELISA

Sandwich ELISA method was employed using ELISA kits to measure the proinflammatory cytokines including IL-6 (ABclonal; Cat: RK00020), TNFα (ABclonal; Cat: RK00029) along with NF-ĸB (ELK Biotechnology; Cat: ELK5691), VEGF-B (FineTest; cat:ER0085) and CCND-1 (FineTest; cat:ER0328). The manufacturers’ recommended procedures were followed, and the spectrophotometric reading was taken at 450 nm.

### mRNA expression

RT-PCR was carried out using the Applied Biosystems 7900HT analyzer (Applied Biosystems, USA). The reaction mixture consisted of 2 µL of cDNA, 10 µL of Sybergreen Master mix (containing 150 mM Tris, pH 9.2, 40 mM (NH4)2SO4, 5 mM MgCl2, 0.02% Tween-20, 0.4 mM dNTPs, 1.25 Units Taq Polymerase, and 1 X Sybergreen), along with 0.5 µL of 20 µM gene-specific primers. TNF-α forward primer: TATGTGCTCTAGGCTAGCTC, reverse primer: GGCATAGTCTCTGCACGTAACT; P53, forward primer: TGAGCCATGAATTGATCATC, reverse primer: AGAAAGACTACCAGAGG; BAX, forward primer: GTTGTGCACATGGCTGGC, reverse primer: CGCATGGTCACTACGCTACCT; Bcl2, forward primer: AGTGTGAACCAGTCGTCTC reverse primer: CGGATTGTCATTGCACTACC; GAPDH, forward primer: TGTGTGCACAAGGCTGGCTC, reverse primer: TGCATGGTCACTGCACTTACCT. To assess gene expression, the threshold cycle (CT) values were measured during the exponential phase of amplification. The ΔCT value was calculated by taking the difference between the CT values of the target genes (TNF-a, Bax, BCL2, and p53) and the CT value of the reference gene GAPDH. Finally, relative quantifications were determined using the formula: 2- ΔCT/ [Average of (2- ΔCT)] [57].

### Histomorphological and immune-histochemical study of the ovary

The ovaries were excised from each animal, and rapidly dissected, cleaned, and weighed. Subsequently, the ovaries were immersed in a 10% formalin solution and stored at 4℃ for HE (hematoxylin and eosin) staining, followed by light microscopic examination. The ovarian tissues were embedded in wax and then sectioned and subjected to HE staining. The follicular growth was evaluated by microscopy.

Immunohistochemistry (IHC) was performed by utilizing a rabbit polyclonal primary antibody targeting the androgen receptor (NR3C4).

### Molecular docking analysis

Molecular docking analysis of Estradiol receptor alpha ligand binding domain (PDB ID- 3ERT) Fig. 9A; androgen receptor ligand binding domain (PDB ID- 2AM9) Fig. 9B and TNFα (PDB ID- 2AZ5) Fig. 9C, protein was performed with the ligand cinnamic acid (PubChem ID- 444,539) Fig. 9A; plumbagin (PubChem ID- 10,205) Fig. 9B and negundin B (PubChem ID-10,473,569) Fig. 9C respectively. PDBQT file format and grid box dimension were prepared before starting the docking process. AutoDock 4.2.6 were used for performing molecular docking and the conformations with lowest binding energy were selected for these three ligands [58]. In this study we preferred the blind docking without any prior active site selection. Biovia discovery studio visualizer and ligplot + software was used for 3D and 2D visualization of protein-ligand interaction.

### Statistical evaluation

The mean ± SE was used to represent the data in this study. The statistical significance was evaluated by two-way ANOVA, followed by post-hoc Tukey test, with a significance level of *p* < 0.05. SPSS software 16.0 was used for statistical analysis.

## Data Availability

Not applicable.

## Abbreviations

VN: *Vitex nigundo*
VNE: *Vitex nigundo* ethanolic extract
PCOS: Polycystic ovarian syndrome
IR: Insulin resistance
LTZ: Letrozole
SOD: Superoxide dismutase
LH: Luteinizing hormone
FSH: Follicular stimulating hormone
NR3C4: Nuclear receptor subfamily 3, group C, member 4
AR: Androgen receptor
ER: α-Estrogen receptor alpha
TNF: α-Tumor necrosis factor alpha
NF: ĸB-nuclear factor kappa-B
IL: 6-Interlukin-6
VEGF: B-Vascular endotheial growth factor-B
CCND1: Cyclin D1
Bcl: 2-B-cell lymphoma 2
BAX: Bcl-2 Associated X protein
